# Catalytic Hydrolysis of Perfluorinated Compounds in a Yolk–Shell Micro‐Reactor

**DOI:** 10.1002/advs.202413203

**Published:** 2025-01-10

**Authors:** Jialin Zheng, Xiaojian Wang, Xin Zi, Hang Zhang, Heping Chen, Evangelina Pensa, Kang Liu, Junwei Fu, Zhang Lin, Liyuan Chai, Emiliano Cortés, Min Liu

**Affiliations:** ^1^ Hunan Joint International Research Center for Carbon Dioxide Resource Utilization School of Physics Central South University Changsha Hunan 410083 P. R. China; ^2^ School of Metallurgy and Environment Central South University Changsha Hunan 410083 P. R. China; ^3^ School of Resource Environment and Safety Engineering University of South China Hengyang Hunan 421001 P. R. China; ^4^ Nanoinstitute Munich Faculty of Physics Ludwig‐Maximilians‐Universität München 80539 München Germany

**Keywords:** catalytic hydrolysis, environmental chemistry, perfluorocarbons, thermal effects, yolk–shell

## Abstract

Perfluorinated compounds (PFCs) are emerging environmental pollutants characterized by their extreme stability and resistance to degradation. Among them, tetrafluoromethane (CF_4_) is the simplest and most abundant PFC in the atmosphere. However, the highest C─F bond energy and its highly symmetrical structure make it particularly challenging to decompose. In this work, a yolk–shell Al_2_O_3_ micro‐reactor is developed to enhance the catalytic hydrolysis performance of CF_4_ by creating a local autothermic environment. Finite element simulations predict that the yolk–shell Al_2_O_3_ micro‐reactor captures the heat released during the catalytic hydrolysis of CF_4_, resulting in a local autothermic environment within the yolk–shell structure that is 50 °C higher than the set temperature. The effectiveness of this local autothermic environment is experimentally confirmed by in situ Raman spectroscopy. As a result, the obtained yolk–shell Al_2_O_3_ micro‐reactor achieves 100% CF_4_ conversion at a considerably low temperature of 580 °C for over 150 h, while hollow and solid Al_2_O_3_ structures required higher temperatures of 610 and 630 °C, respectively, to achieve the same conversion rate, demonstrating the potential of yolk–shell Al_2_O_3_ micro‐reactor to significantly reduce the energy requirements for PFCs degradation and contribute to more sustainable and effective environmental remediation strategies.

## Graphical abstract

The yolk–shell micro‐reactor captures the heat released during the catalytic hydrolysis of tetrafluoromethane (CF_4_) to create a local autothermic environment between the yolk and the shell, increasing the local temperature by 50 °C. As a result, the yolk–shell micro‐reactor achieved 100% CF_4_ degradation at a considerably low temperature of 580 °C for over 150 h.

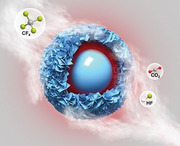

## 1 Introduction

Perfluorinated compounds (PFCs) are among the most persistent environmental pollutants, known for their extreme resistance to decompose, which has garnered global attention.^[^ ^1^, ^2^, ^3^, ^4^, ^5^, ^6^, ^7^, ^8^ ^]^ Tetrafluoromethane (CF_4_), the simplest PFCs, features highly symmetrical C─F with a bond energy of up to 543 ± 4 kJ mol^−1^, and an atmospheric lifetime exceeding 50 000 years, making it exceptionally challenging to break down naturally.^[^ ^9^, ^10^, ^11^, ^12^, ^13^, ^14^ ^]^ Therefore, developing an efficient method for CF_4_ catalytic hydrolysis is highly desirable for a sustainable future.

Thermocatalytic hydrolysis is regarded as one of the most effective techniques for CF_4_ treatment, but its high‐temperature requirements and significant energy consumption pose substantial challenges for industrial applications.^[^ ^15^, ^16^, ^17^, ^18^, ^19^, ^20^, ^21^, ^22^ ^]^ To enhance catalytic activity and reduce energy consumption, researchers are actively optimizing the Al_2_O_3_ catalyst.^[^ ^23^, ^24^, ^25^, ^26^, ^27^, ^28^, ^29^, ^30^, ^31^ ^]^ For example, Zhang et al. reported the preparation of various crystalline phases of Al_2_O_3_, in which γ‐Al_2_O_3_ could degrade CF_4_ 100% at 650 °C.^[^ ^22^ ^]^ EI‐Bahy et al. found that a Ga‐Al catalyst could achieve 100% conversion of CF_4_ at 630 °C.^[^ ^23^ ^]^ Despite these results demonstrating the efficient CF_4_ conversion, the high temperatures required resulted in significant energy consumption. Recently, Lai et al. discovered that utilizing thermal cycling within a porous media structure can improve the reaction conversion rate and reduce energy consumption.^[^ ^32^ ^]^ Consequently, energy consumption during hydrolysis can be minimized by precisely adjusting the catalyst's local autothermic environment to harness the energy released by CF_4_ conversion, an exothermic reaction that releases a large amount of energy (168 KJ mol^−1^).^[^ ^33^, ^34^, ^35^, ^36^ ^]^

Herein, we developed a yolk–shell Al_2_O_3_ micro‐reactor to create a local autothermic environment, thereby enhancing the catalytic hydrolysis performance of CF_4_. Finite element simulations using COMSOL demonstrated that the yolk–shell Al_2_O_3_ catalyst can effectively utilize the heat released by CF_4_ catalytic hydrolysis to establish a local autothermic environment, increasing the reaction temperature by 50 °C. In contrast, hollow and solid Al_2_O_3_ catalysts could only achieve temperature increases of 15 and 0 °C, respectively, under the same conditions. The successful preparation of yolk–shell, hollow, and solid structured catalysts was confirmed through scanning electron microscopy (SEM) and transmission electron microscopy (TEM). X‐ray powder diffraction (XRD) analyses proved that all catalysts were indeed Al_2_O_3_. Furthermore, in situ Raman spectroscopy experimentally confirmed the generation of a local autothermic environment. The catalytic hydrolysis performance tests showed that the yolk–shell Al_2_O_3_ micro‐reactor achieved a 100% CF_4_ conversion rate at 580 °C for over 150 h, whereas hollow and solid Al_2_O_3_ catalysts required temperatures of 610 and 630 °C, respectively, to achieve the same conversion rate. This work presents a novel catalyst design that effectively harnesses reaction heat to create a local autothermic environment, offering a new strategy for the low‐temperature, high‐efficiency catalytic hydrolysis of PFCs.

## 2 Results and Discussion

### 2.1 Finite Element Simulations

**Figure 1:**
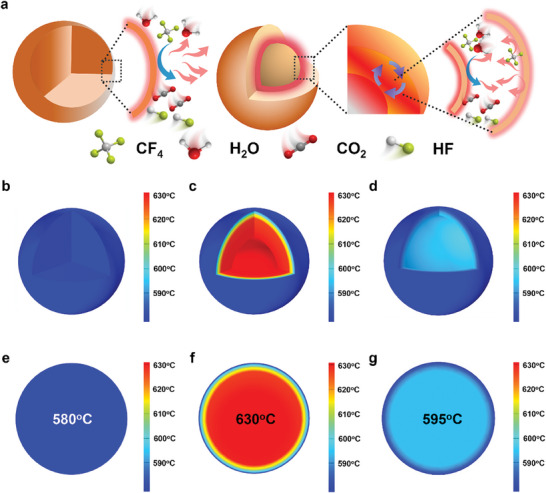
Finite element simulations. a) Schematic illustration of the direction of heat generated by catalytic hydrolysis of CF_4_ in different catalyst structures. COMSOL simulates the temperature distribution for b,e) solid, c,f) yolk–shell, and d,g) hollow Al_2_O_3_ catalysts during the catalytic hydrolysis of CF_4_.

To validate the feasibility of constructing a local autothermic environment utilizing the heat released by CF_4_ catalytic hydrolysis reaction, we conducted simulations using COMSOL for yolk–shell, hollow, and solid Al_2_O_3_ catalysts (**Figure** **1**a).^[^ ^37^, ^38^, ^39^, ^40^ ^]^ Spherical models of these catalysts were constructed to simulate the temperature distributions during the catalytic hydrolysis of CF_4_. Using 580 °C as the baseline catalytic hydrolysis temperature, the simulation revealed that the yolk–shell Al_2_O_3_ catalyst exhibited a higher internal temperature of 630 °C within its cavity (Figure 1c–f), while the hollow Al_2_O_3_ catalyst only reached an internal temperature of 595 °C (Figure 1d–g). In contrast, the solid Al_2_O_3_ shows no significant temperature difference (Figure 1b–e). These indicate that the yolk–shell Al_2_O_3_ catalyst can utilize the energy released during CF_4_ catalytic hydrolysis to create a local autothermic environment, raising the local temperature by 50 °C.

### 2.2 Synthesis and Characterizations of Catalysts

**Figure 2:**
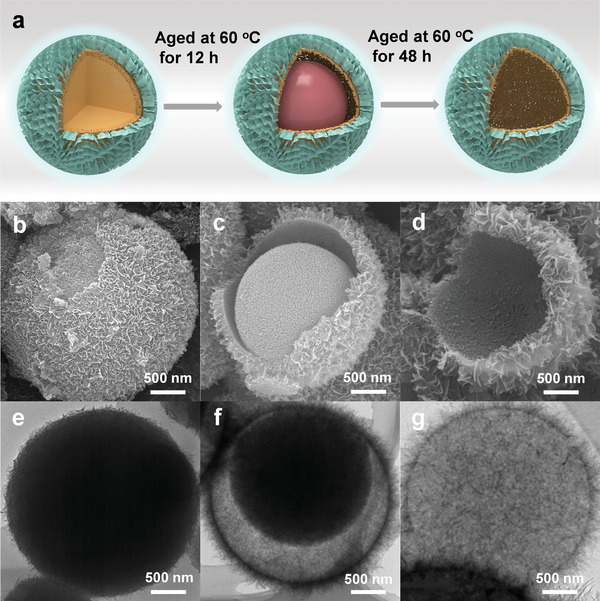
Characterization of yolk–shell, hollow, and solid catalysts. a) Schematic illustration of the preparation process for three catalysts. b–d) SEM images of yolk–shell, hollow, and solid Al_2_O_3_ catalysts respectively. e–g) TEM images of yolk–shell, hollow, and solid Al_2_O_3_ catalysts respectively.

To validate the simulation prediction, a yolk–shell Al_2_O_3_ catalyst was synthesized by a sol‐gel method with aging time adjustment (**Figure** **2**a). X‐ray diffraction (XRD, Figure S1, Supporting Information) confirmed that the obtained yolk–shell, hollow, and solid Al_2_O_3_ all correspond to the γ‐Al_2_O_3_ phase (PDF#79‐1558). Scanning electron microscopy (SEM) images showed that the prepared catalysts exhibited a sea urchin‐like morphology, with an external surface composed of randomly assembled and interconnected nanosheets (Figure 2b–d and S2, Supporting Information). Transmission electron microscopy (TEM) images further revealed that the yolk–shell, hollow, solid Al_2_O_3_ catalysts had diameters in the range of 2–3 µm, with the yolk in the yolk–shell structure measuring ≈ 1.5 µm, consistent with SEM results (Figure 2e–g). Energy‐dispersive spectroscopy (EDS) mapping illustrated uniform distributions of Al and O across the catalyst (Figure S3, Supporting Information). Al *2p* X‐ray photoelectron spectroscopy (XPS) spectra demonstrated the chemical valence states of Al in the yolk–shell, hollow, and solid Al_2_O_3_ catalysts are consistent (Figure S4, Supporting Information). Brunauer–Emmett–Teller (BET) analyses revealed that the specific surface areas of yolk–shell, hollow, and solid Al_2_O_3_ catalysts were 305.3, 331.4, and 283.6 m^2^ g^−^¹, respectively (Figure S5 and Table S1, Supporting Information), excluding the effect of specific surface area. These results confirmed the successful preparation of yolk–shell, hollow, and solid Al_2_O_3_ catalysts with identical physical and chemical properties.

### 2.3 Local Thermal Field Effect

**Figure 3:**
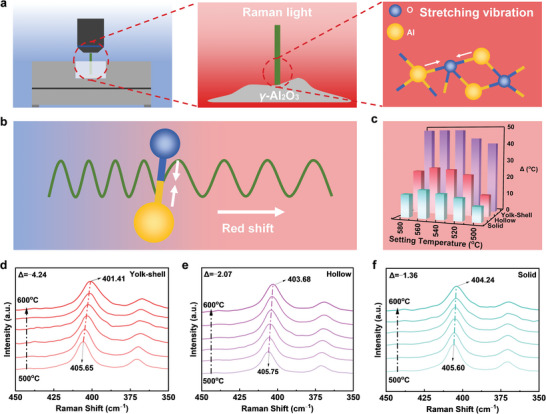
Local thermal field effect. a,b) Schematic diagram of Raman test mechanism. c) The difference between the actual temperature and the setting temperature of yolk–shell, hollow, and solid Al_2_O_3_ catalysts is calculated by fitting. d–f) Shift of Raman peak with increasing temperature during a reaction of yolk–shell, hollow, and solid Al_2_O_3_ catalysts.

To experimentally validate the creation of a local thermal environment, we employed in situ Raman spectroscopy to monitor temperature changes of the catalyst during CF_4_ catalytic hydrolysis (**Figure** **3**a; Figure S6, Supporting Information).^[^ ^41^ ^]^ During the CF₄ catalytic hydrolysis reaction, the tensile vibration of the Al─O bond is affected by temperature changes, resulting in a shift of the Raman peak toward lower wavenumbers (Figure 3b). The results showed that the Raman peak of the yolk–shell Al_2_O_3_ catalyst shifted from 405.7 to 401.4 cm^−^¹, corresponding to an offset of 4.3 cm^−^¹. In comparison, the offsets for the hollow and solid Al_2_O_3_ catalysts were only 2.1 and 1.4 cm^−^¹, respectively (Figure 3d–f; Figure S7, Supporting Information).

Based on the linear relationship between Raman frequency shift and temperature,^[^ ^42^ ^]^ the actual temperatures could be calculated, allowing us to determine the difference (Δ°C) between the set and actual temperatures (Figure 3c). As shown in the figure, when the external temperature was set to 580 °C, the actual temperature in the yolk–shell catalyst reached 627 °C, resulting in a Δ°C of 47 °C. In contrast, the hollow and solid catalysts exhibited Δ°Cs of 24 and 12 °C, respectively. These Δ°Cs demonstrated that the yolk–shell catalyst has a superior capacity to accumulate and retain heat compared to the hollow and solid structures, thereby creating a localized thermal field during the catalytic process.

### 2.4 CF_4_ Catalytic Hydrolysis Performance

**Figure 4:**
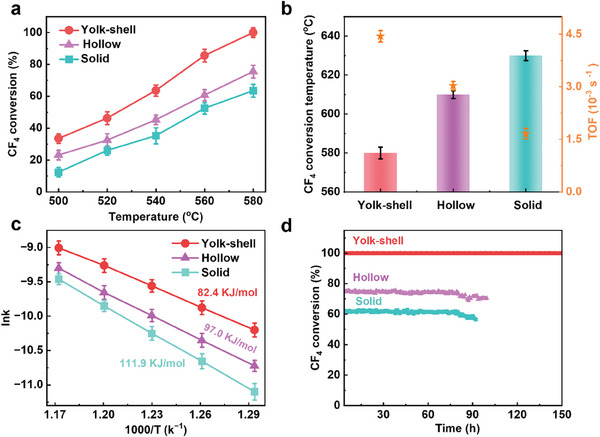
Catalytic stability and performance characterizations. a) CF_4_ conversion (%) during CF_4_ catalytic hydrolysis reaction at different reaction temperatures for yolk–shell, hollow, and solid Al_2_O_3_ catalysts. b) Temperature at 100% catalytic hydrolysis of the three samples and TOF calculation for catalytic conversion of CF_4_. c) Arrhenius plots obtained for the CF_4_ conversion rates at 500–580 °C for the three catalysts. d) The stability test of yolk–shell, hollow, and solid Al_2_O_3_ catalysts at 580 °C. (Reaction condition: 2500 ppm of CF_4_ and 10% of H_2_O, balanced with Ar, total flow rate of 33.3 mL min^−1^, and weight hourly space velocity (WHSV) of 1000 mL g ^−1^ h^−1^).

To demonstrate the effectiveness of the local autothermal environment, we evaluated the CF_4_ hydrolysis activities of yolk–shell, hollow, and solid Al_2_O_3_ catalysts over a temperature range of 500 –580 °C (**Figure** **4**a; Figure S8, Supporting Information). Through this temperature range, the yolk–shell Al_2_O_3_ catalyst consistently exhibited higher CF_4_ conversion rates than those for the hollow and solid Al_2_O_3_ catalysts, further confirming the effectiveness of the local autothermal environment. As a result, the yolk–shell Al_2_O_3_ catalyst achieved 100% CF_4_ conversion at 580 °C, while temperatures of 610 and 630 °C are required to achieve the same conversion rates over hollow and solid Al_2_O_3_ catalysts, respectively (Figure 4b). This significant reduction in required temperature underscores the effectiveness of the yolk–shell structure in enhancing catalytic performance.

To rule out potential influences from the number and properties of acidic sites or CF_4_ adsorption capabilities, temperature‐programmed desorption of NH_3_ (NH_3_‐TPD), pyridine‐infrared (py‐IR), and CF_4_‐TPD was conducted. The NH_3_‐TPD and Py‐IR analyses indicated that only L‐acid sites on the catalyst surfaces (Figures S9 and S10, Supporting Information). CF_4_‐TPD results showed that the CF_4_ adsorption capacity of hollow Al_2_O_3_ was higher than those of yolk–shell and solid Al_2_O_3_ (Figure S11, Supporting Information), indicating that the enhancement of CF_4_ conversion over yolk–shell Al_2_O_3_ was not caused by its adsorption capacity but its local autothermic nature.

To investigate the intrinsic activity of the catalysts, the turnover frequency (TOF) of the catalysts was calculated at 500 °C based on surface acidity test results. The yolk–shell Al_2_O_3_ catalyst exhibited a TOF of 4.4 × 10^−^^3^ s^−^¹, which was 1.5 and 2.7 times higher than those of hollow and solid Al_2_O_3_ catalyst, respectively (Figures S12 and Table S2, Supporting Information). The calculated apparent activation energies for the yolk–shell, hollow, and solid Al_2_O_3_ catalysts are 84.2, 97.0, 111.9 kJ mol^−^¹, respectively (Figure 4c; Figures S8 and S13, Supporting Information), further proving the enhanced catalytic activity by the local autothermic environment created in the yolk–shell structure.

Next, the stability of the catalysts was assessed at 580 °C. The results showed that the yolk–shell Al_2_O_3_ catalyst maintained a 100% CF_4_ conversion for over 150 h (Figure 4d; Figure S14, Supporting Information), significantly outperforming 75% CF_4_ conversion for 80 h and 60% CF_4_ conversion for 75 h over hollow and solid Al_2_O_3_ respectively. These results demonstrate that the yolk–shell Al_2_O_3_ catalyst offers both superior catalytic activity and stability, and has certain advantages compared with the reported catalyst (Table S3, Supporting Information).

In order to understand the different stability of the catalysts, we performed XRD and SEM characterizations of the catalysts after a long reaction time. The XRD results show that all the catalysts change from the γ‐Al_2_O_3_ phase to the inactive α‐Al_2_O_3_ phase, accounting for the decrease in catalyst stability (Figure S15, Supporting Information). In addition, during CF_4_ catalytic hydrolysis, fluorine is adsorbed on the Al active sites, resulting in catalyst fluorine poisoning and reduced stability. The higher temperature is conducive to breaking the Al─F bonds, realizing the regeneration of the Al active sites, and improving the stability of the catalyst. Finite element simulations and in situ Raman spectroscopy indicate that the yolk–shell Al_2_O_3_ catalyst creates the local autothermic environment, which makes the cavity temperature higher than that of hollow and solid Al_2_O_3_ catalysts, and is conducive to the regeneration of the Al active sites (Figure S16a, Supporting Information). Although the hollow Al_2_O_3_ catalyst also has a local autothermic environment (weaker than the yolk–shell Al_2_O_3_), the hollow Al_2_O_3_ catalyst is more prone to collapse, hindering the regeneration of the Al active sites, so the activity of the hollow Al_2_O_3_ catalyst begins to decline at 80 h (Figure S16b, Supporting Information). The solid structure lacks a local autothermic environment, so the stability decreases due to the fluorine poisoning of the Al active sites after a 75 h reaction (Figure S16c, Supporting Information).

## 3 Conclusion

In summary, we developed a yolk–shell Al_2_O_3_ micro‐reactor catalyst that leverages the cavity between the yolk and shell to trap the heat released during the reaction, creating a localized autothermic environment that enhances CF_4_ catalytic hydrolysis. COMSOL simulations demonstrated that the yolk–shell Al_2_O_3_ catalyst effectively utilizes the heat from CF_4_ catalytic hydrolysis to establish a local autothermic environment, raising the reaction temperature by 50 °C. In contrast, hollow and solid Al_2_O_3_ catalysts exhibited much smaller temperature increases of 15 and 0 °C, respectively, under the same conditions. In situ Raman spectroscopy confirmed the creation of this localized autothermic environment. Performance tests revealed that the yolk–shell Al_2_O_3_ catalyst achieved 100% CF_4_ conversion at 580 °C and maintained this activity for over 150 h. This work introduces a novel approach for designing low‐temperature, high‐activity catalysts for the catalytic hydrolysis of PFCs.

## Conflict of Interest

The authors declare no conflict of interest.

## Supporting information



Supporting Information

## Data Availability

The data that support the findings of this study are available from the corresponding author upon reasonable request.
