# Supplementary material for: Catalytic Hydrolysis of Perfluorinated Compounds in a Yolk–Shell Micro‐Reactor
Source: Adv Sci (Weinh). 2025 Jan 10;12(9):2413203. doi: 10.1002/advs.202413203 (PMC11884611; doi:10.1002/advs.202413203)
Supplement: Supplementary file 1 — Supporting Information [file ADVS-12-2413203-s001.docx]

**Supporting Information**

**Catalytic Hydrolysis of Perfluorinated Compounds in Yolk-shell Micro-reactor**

Jialin Zheng^+, [a, b]^, Xiaojian Wang^+, [a, b]^, Xin Zi ^[a]^, Hang Zhang ^[a]^, Heping Chen ^[c]^, Evangelina Pensa ^[d]^, Kang Liu ^[a]^, Junwei Fu ^[a]^, Zhang Lin ^[b]^, Liyuan Chai ^[b]^, Emiliano Cortés ^[d],^ * and Min Liu ^[a, b],^ *

[a] J. Zheng, X. Wang, X. Zi, H. Zhang, Dr. K. Liu, Prof. J. Fu, and Prof. M. Liu
Hunan Joint International Research Center for Carbon Dioxide Resource Utilization
School of Physics
Central South University
Changsha 410083, Hunan, P.R. China
E-mail: [minliu@csu.edu.cn](mailto:minliu@csu.edu.cn)

[b] J. Zheng, X. Wang, Prof. Z. Lin, Prof. L. Chai, and Prof. M. Liu
School of Metallurgy and Environment
Central South University
Changsha 410083, Hunan, P.R. China

[c] H. Chen
School of Resource Environment and Safety Engineering
University of South China
Hengyang 421001, Hunan, P. R. China

[d] Dr. E. Pensa and Prof. E. Cortés
Nanoinstitute Munich, Faculty of Physics
Ludwig-Maximilians-Universität München
80539, München, Germany
E-mail: [Emiliano.Cortes@lmu.de](mailto:Emiliano.Cortes@lmu.de)

[+] These authors contributed equally to this work.

**Experimental Section**

**Materials and Methods**

All chemicals were obtained commercially and used as received. Aluminum chloride hexahydrate (AlCl_3_·6H_2_O, 97%), Propylene oxide (C_3_H_6_O, 99%) and anhydrous ethanol (CH_3_CH_2_OH, ≤0.3%) were purchased from Aladdin. Deionized water was used in all experiments.

**Catalyst Preparation**

First, 30 mmol AlCl_3_·6H_2_O was dissolved in the mixture solvent of deionized water and anhydrous ethanol (6 ml: 9 ml) by stirring at 60 ^o^C. Then, 10.5 ml of propylene oxide was added into the mixture solution. The resulting solution was sealed and kept at 60 ^o^C for gelation and immerse the sample in anhydrous ethanol at 60 ^o^C for different time. Finally, calcine the gel precursor at 600 ^o^C for 3 hours.

**Catalytic Reaction System**

The quartz fixed bed reactor continuous flow reaction system was used to catalyze the hydrolysis of CF_4_, and the reaction temperature range was 500 to 600 ^o^C. The gas flow rate of 33.3 mL min^-1^ (0.25% CF_4_ in Ar) was controlled by the mass flow controller, the 0.8 mL h^-1^ water is introduced by an injection pump, vaporized in a gasification chamber, and 2.0 g catalyst was loaded into the reactor.

$$\text{CF}_{\text{4}}\text{ decomposition}\left( \text{\%} \right)\text{=}\frac{{\text{[}\text{CF}_{\text{4}}\text{]}}_{\text{in}}\text{-}{\text{[}\text{CF}_{\text{4}}\text{]}}_{\text{out}}}{{\text{[}\text{CF}_{\text{4}}\text{]}}_{\text{in}}}\text{×100\%}$$

Where [CF_4_]_in_ and [CF_4_]_out_ indicate the inlet and outlet relative concentrations, respectively.

**Catalyst Characterization**

X-ray powder diffraction (XRD) was measured by a Bruker D8 Focus diffractometer with Cu Kα (40 kV, 40 mA) radiation as the X-ray source for scanning in the 2θ range of 10 to 90°.

Transmission electron microscopy (JEOL 3010, 200 kV) and Scanning electron microscopy (MIRA3 LMH, 20.0 kV) to observed the morphology of the samples. The sample was dispersed in ethanol solution and then dropped on copper grid before TEM, it is essential to coat the sample with Au prior to the analysis for SEM imaging.

X-ray photoelectron spectroscopy (XPS) was performed using a Kratos Axis Ultra DLD spectrometer with a standard Al Kα X-ray source and an analyzer pass energy of 40 eV. All binding energies were referenced to the adventitious C *1s* peak at 284.6 eV.

N_2_ adsorption-desorption analysis was performed using the Micromeritics ASAP 2460 to determine the surface area and pore size distribution of the catalysts. Prior to the measurement, the sample was degassed at 300 ^o^C for 6 hours.

Temperature programmed desorption of NH_3_ (NH_3_-TPD) was measured with PCA-1200 chemical absorption analyzer equipped with TCD detector. To remove surface impurities and moisture from the sample, pretreat 0.1 g of the sample in high-purity Ar gas at 600 ^o^C for 2 hours. Subsequently, switch the gas to NH_3_ for 1 hour, followed by purging with Ar gas for 1 hour at room temperature. Upon removing the physically adsorbed NH_3_, begin heating at a rate of 10 ^o^C min^-1^ up to 600 ^o^C.

*In situ* Raman tests were carried out on a DXR3 Raman Microscope equipped with a 532 nm excitation source, and wavenumbers in the range of 3000 ~ 100 cm^−1^. Prior to each test, the powder sample was compacted, loaded into an *in situ* Raman sample cell and purged for 1 h with Ar gas (33.3 mL min^−1^) at the target temperature. The corresponding spectra were recorded as unreacted spectra.

*In situ* Raman spectra of the CF_4_ reaction were obtained followed a similar protocol. Before recording the spectra, the catalysts were maintained at each temperature for 1 h with a mixture flow of CF_4_ and Ar (2500 ppm of CF_4_ balanced with Ar, total flow rate of 33.3 mL min^−1^). The Raman spectra of the solid, yolk-shell, and hollow catalysts in Ar and Ar + CF_4_ atmospheres were obtained at a temperature range of 500 to 600 °C with 20 °C intervals.

Pyridine-infrared (py-IR) spectra of samples were analyzed by a Thermo IS-50 Fourier Transform infrared (FTIR) spectrometer. The sample was placed in a vacuum oven and vacuumized to 10^−3^ Torr, samples were exposed to pyridine vapor (3,000 Pa) at 100 °C for 1 h, followed by re-evacuation for 1 h, and lower the temperature to take out our samples. After this step, the sample was analyzed by FTIR.

**Simulation Section**

**COMSOL simulations:** The finite element method (FEM) simulations were conducted through COMSOL Multiphysics v 6.0. The Heat transfer (HT) physics modules in COMSOL Multiphysics were used to simulate and calculate the performance of the models. Modeling the catalyst particles using experimental images. All the meshes in the model were set to free tetrahedral meshing. The relative tolerance in the steady-state solver was set to 0.0001.

Considering both heat conduction and convection, the specific formula is:


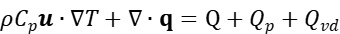


where $C_{p}$ is the heat capacity at constant pressure, $T$ is the thermodynamic temperature, $\rho$ is the material density, $\boldsymbol{u}$ is the velocity vector, $Q$ , $Q_{p}$ and $Q_{vd}$ are the heat source terms, $\nabla\cdot\mathbf{q}$ is the divergence of the temperature gradient. It can be obtained from the following formula:

$$\mathbf{q}=-k_{iso}\nabla T$$

Where $k_{iso}$ is the thermal conductivity of the material.

The gas thermal conductivity is corrected using the following formula:

$$k_{iso,\gamma-{Al}_{2}O_{3}}=-0.00227583562+1.15480022\times{10}^{-4}\times T-7.90252856\times{10}^{-8}\times T^{2}+4.11702505\times{10}^{-11}\times T^{3}-7.43864331\times{10}^{-15}*T^{4}$$

The density of the catalyst particles is corrected using the following formula:

$$\rho_{\gamma-{Al}_{2}O_{3}}=\left( 3726.112-0.0896537*T \right)$$

The boundary conditions for the inflow and outflow of the gas flow field are described by the following equations:

$$-\boldsymbol{n}\cdot\mathbf{q}=\rho\Delta H\boldsymbol{u}\cdot\boldsymbol{n}$$

and

$$\Delta H=\int_{T_{ustr}}^{T} C_{p}dT$$

where $\boldsymbol{n}$ is the direction vector. The boundary temperature is set to the reaction temperature $T_{0}$.

The gas flow rate through the catalyst particles is corrected by the porosity $\varepsilon_{i}$ and is calculated in conjunction with the reactor radius:

$${flow rate}_{total}={flow rate}_{air+{CF}_{4}}+{flow rate}_{water}=\frac{TP_{0}*33.3 sccm}{T_{0}P}+\left( \frac{0.06 g}{M_{H_{2}O}}*\frac{RT}{P}*{10}^{6} \frac{{cm}^{3}}{m^{3}} \right) s^{-1}$$

and

$$v_{total}=\frac{{flow rate}_{total}}{\pi{r_{reactor}}^{2}(\varepsilon_{i})}$$

where $r_{reactor}$ is the reactor radius. $\varepsilon_{i}$ is estimated based on the experimental bulk density of the catalyst:

$$\varepsilon_{i}=\frac{\rho_{bulk i}}{\rho_{\gamma-{Al}_{2}O_{3}}}$$

Considering exothermic reactions:

$${CF}_{4}+{2H}_{2}O\to{CO}_{2}+4HF (\Delta H=-168.1 kJ {mol}^{-1})$$

where $\Delta H$ is calculated based on bond energy and is temperature-corrected using the heat capacity at constant pressure.

The heat generated by the catalyst particles is estimated using the following formula:

$$Q_{total}={flow rate}_{air+{CF}_{4}}\times0.25\%\times\frac{RT}{P}\times\Delta H$$

and

$$Q_{i}=\frac{Q_{total}}{n_{i}}$$

where ${flow rate}_{air+{CF}_{4}}\times0.25\%$ represents the gas flow rate entering the reactor under operating conditions and the proportion of CF_4_. where $n_{i}$ denotes the number of catalyst particles estimated.

Model parameters of COMSOL simulations.

| **Designation** | **Value** |
| --- | --- |
| $k_{iso,\gamma-{Al}_{2}O_{3}}$ | $11.8 W m^{-1}K^{-1}$ |
| $n_{yolk shell}$ | $2.396{*10}^{13} {kg}^{-1}$ |
| $n_{solid}$ | $5.396{*10}^{13} {kg}^{-1}$ |
| $n_{hollow}$ | $1.2111{*10}^{13} {kg}^{-1}$ |
| $M_{{Al}_{2}O_{3}}$ | $0.10196 kg {mol}^{-1}$ |
| $M_{H_{2}O}$ | $0.0180153 kg {mol}^{-1}$ |
| $r_{solid}$ | $967.5 nm$ |
| $r_{hollow outer}$ | $1613 nm$ |
| $r_{hollow inner}$ | $1363 nm$ |
| $\rho_{bulk yolk shell}$ | $102.6 kg m^{-3}$ |
| $\rho_{bulk solid}$ | $205.4 kg m^{-3}$ |
| $\rho_{bulk shell}$ | $104.9\mathrm{kg}m^{-3}$ |
| $C_{p,air}$ | $1.004 J g^{-1} K^{-1}$ |
| $r_{reactor}$ | $1 cm$ |
| $\varepsilon_{yolk shell}$ | $0.763$ |
| $\varepsilon_{solid}$ | $0.7924$ |
| $\varepsilon_{hollow}$ | $0.6198$ |
| $T_{0}$ | $853 K$ |
| *P_0_* | *1 atm* |

**Additional Figures**


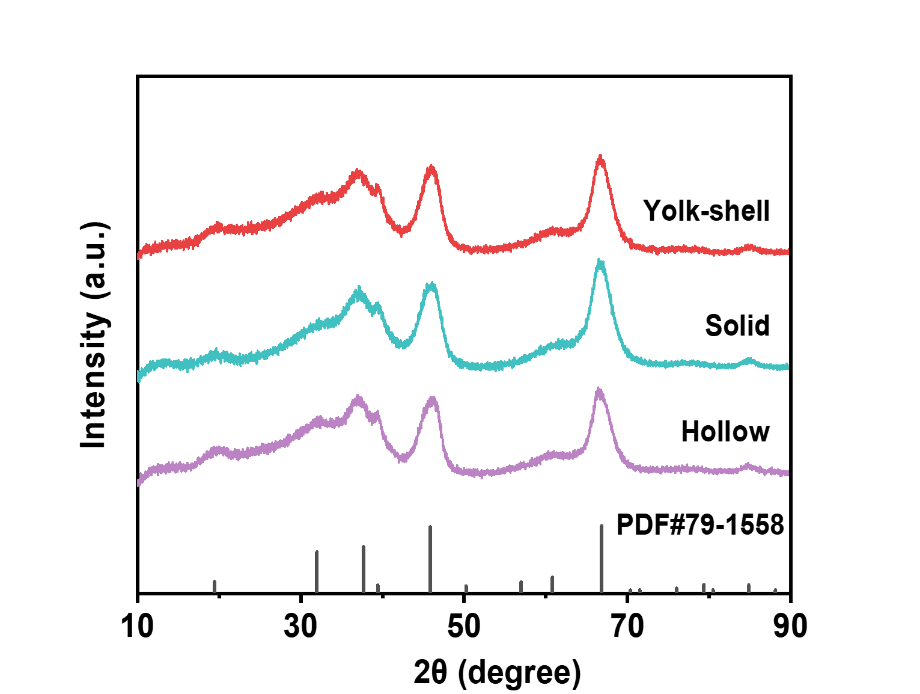


**Figure S1.** XRD patterns of the yolk-shell, hollow, solid Al_2_O_3_ catalyst.

**Figure S2.** SEM images of yolk-shell, hollow and solid Al_2_O_3_ catalysts.

**Figure S3.** EDS mapping of (a – c) solid, (d – f) yolk-shell and (g – i) hollow Al_2_O_3_ catalysts.

**Figure S4.**XPS spectra of Al *2p* for yolk-shell, hollow and solid Al_2_O_3_ catalysts.


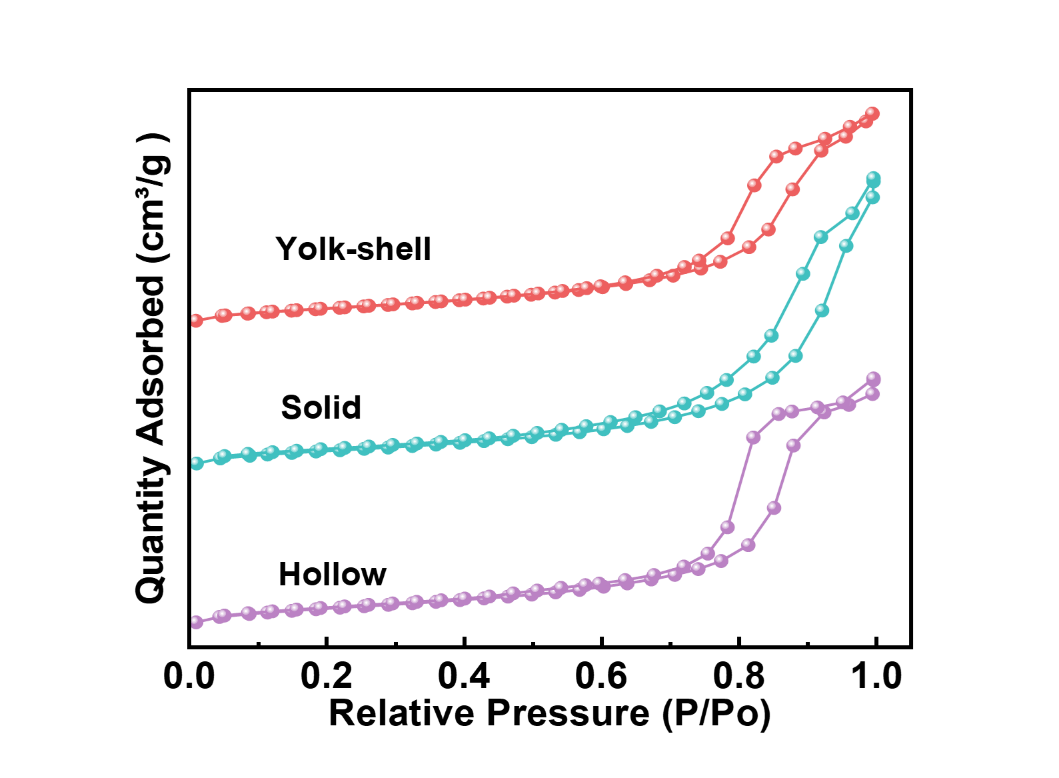


**Figure S5.** N_2_ adsorption–desorption isotherms of yolk-shell, hollow and solid Al_2_O_3_ catalysts.

**Figure S6.** Thermal catalysis *in situ* Raman diagram.

**Figure S7.** (a - c) Frequency shift of Raman peak and the linear curve of temperature before and after reaction of yolk-shell, hollow and solid Al_2_O_3_ catalysts. (d) The relationship between the setting temperature of yolk-shell, hollow and solid Al_2_O_3_ catalysts and the actual temperature.


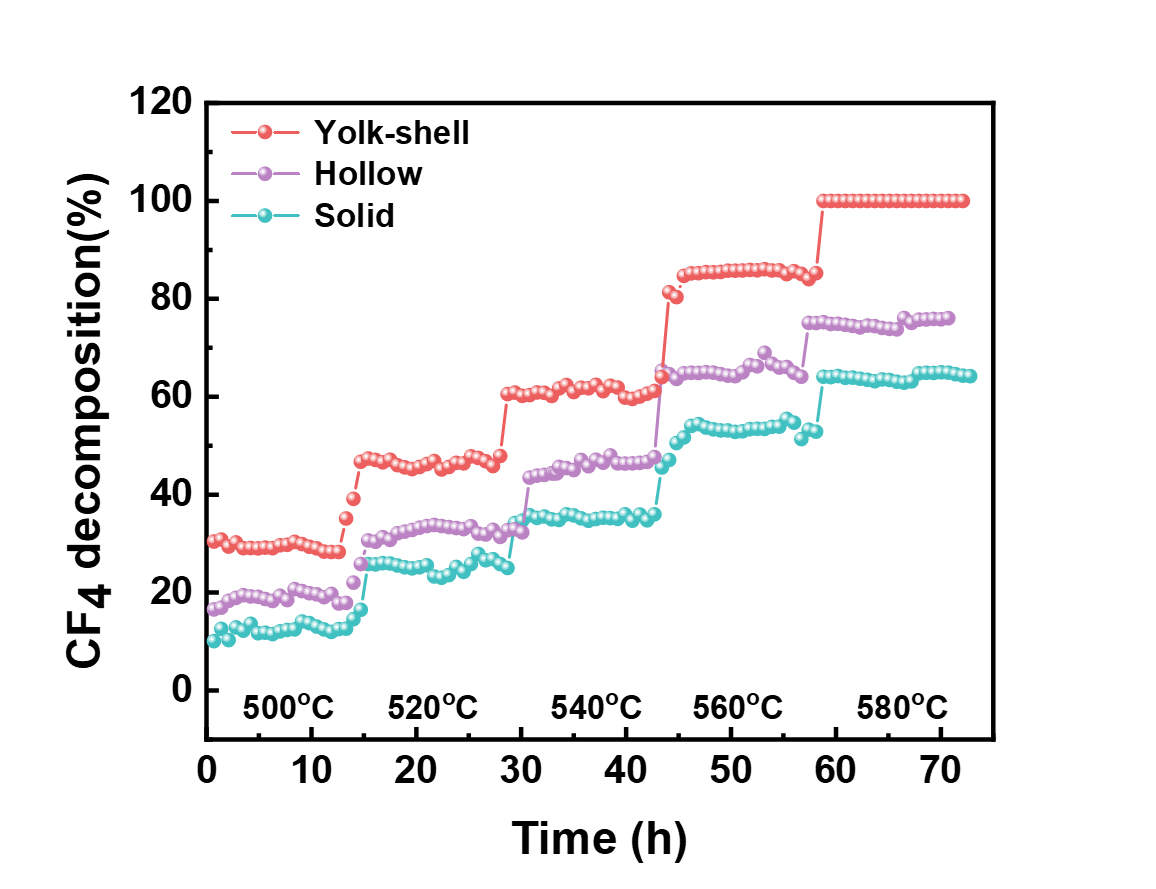


**Figure S8.** CF_4_ decomposition (%) during CF_4_ catalytic hydrolysis reactions at different reaction temperatures over yolk-shell, hollow and solid Al_2_O_3_ catalysts.


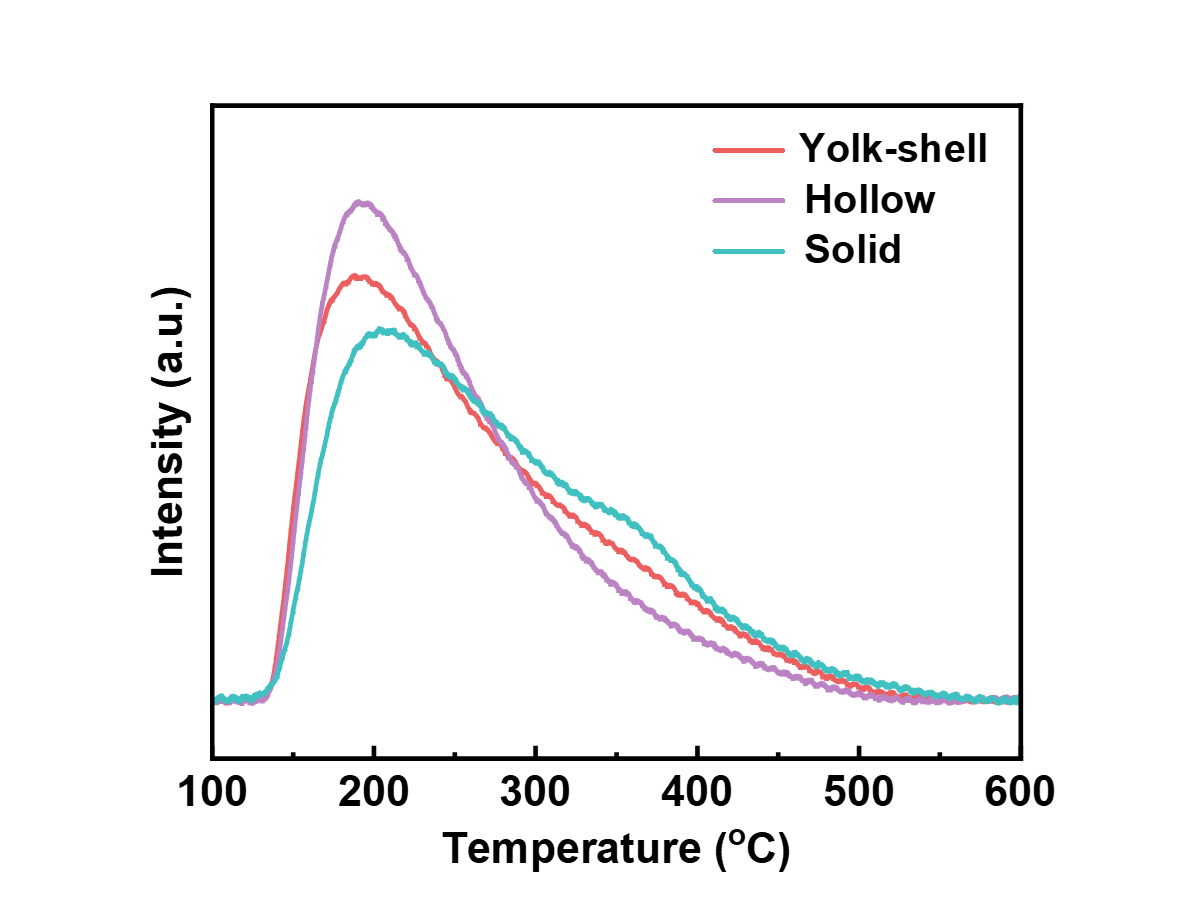


**Figure S9.** NH_3_-TPD profiles of the yolk-shell, hollow and solid Al_2_O_3_ catalysts.


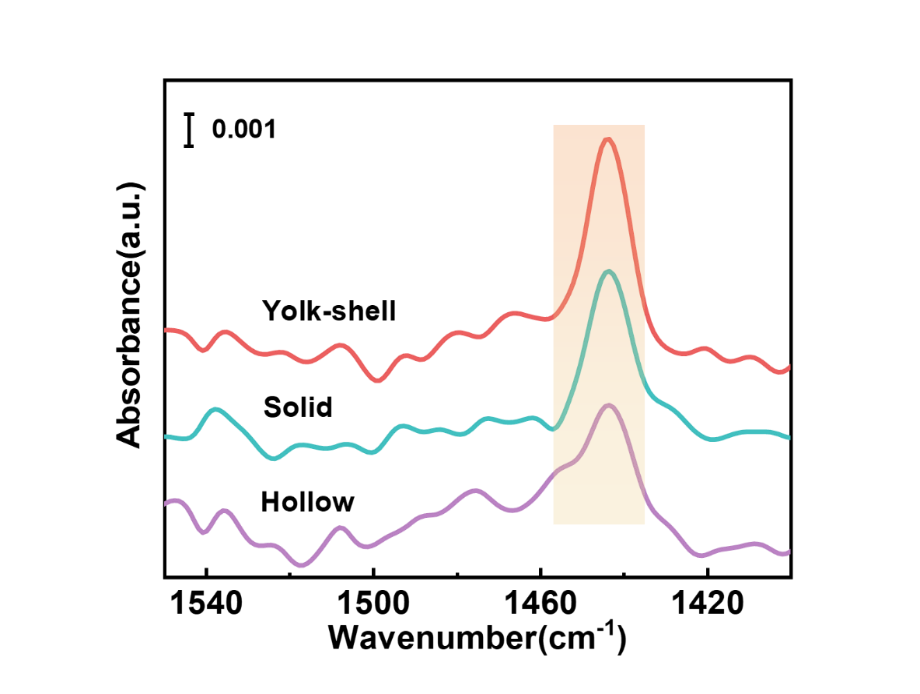


**Figure S10.** Py-IR spectra of the yolk-shell, hollow and solid Al_2_O_3_ catalysts at 100 ^o^C desorption temperature.


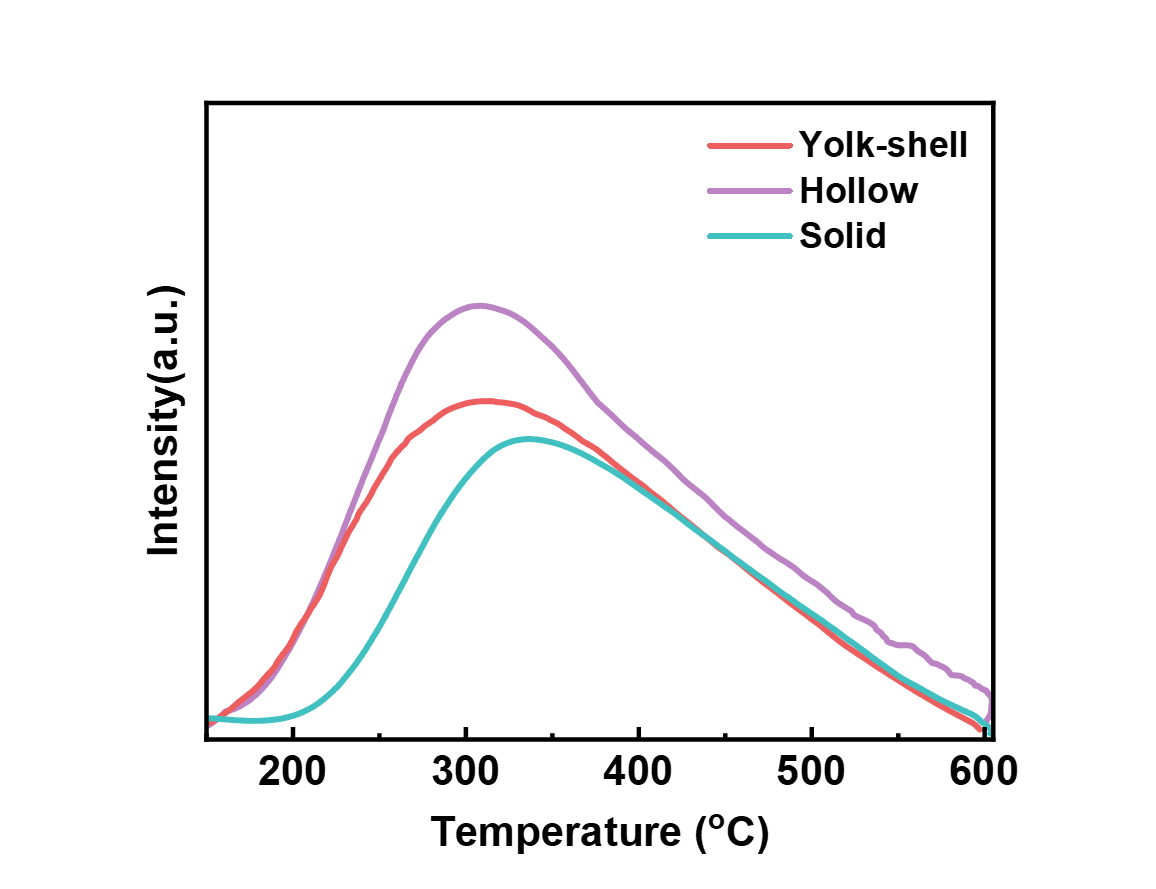


**Figure S11.** CF_4_-TPD of yolk-shell, hollow and solid Al_2_O_3_ catalysts.

**Figure S12.** TOF calculation of CF_4_ catalytic decomposition by yolk-shell, hollow and solid Al_2_O_3_ catalysts.


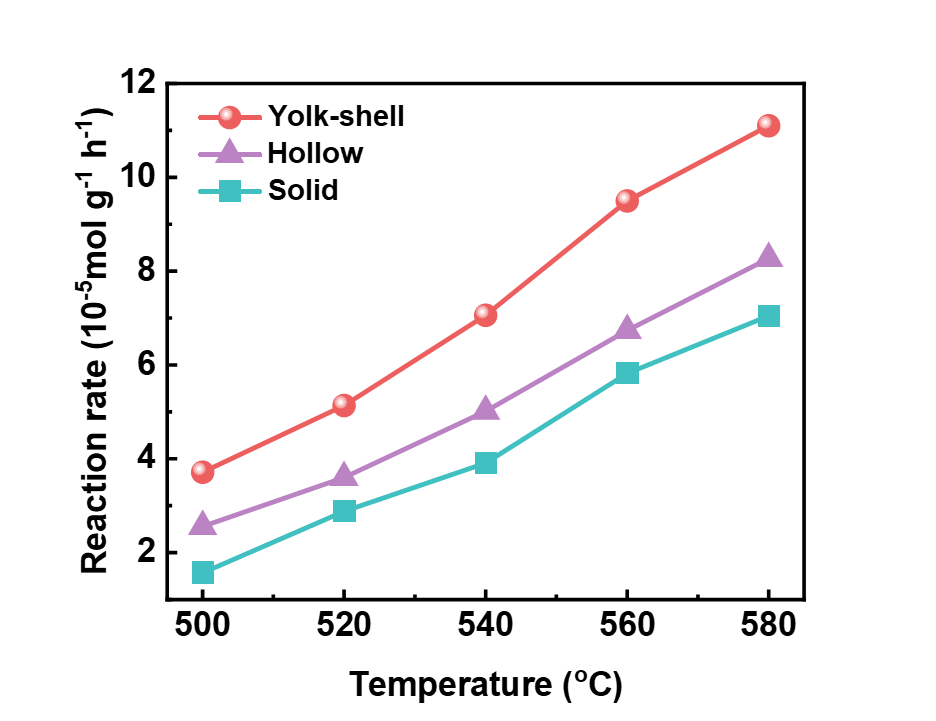


**Figure S13.** Reaction rate of yolk-shell, hollow and solid Al_2_O_3_ catalysts at different reaction temperatures.


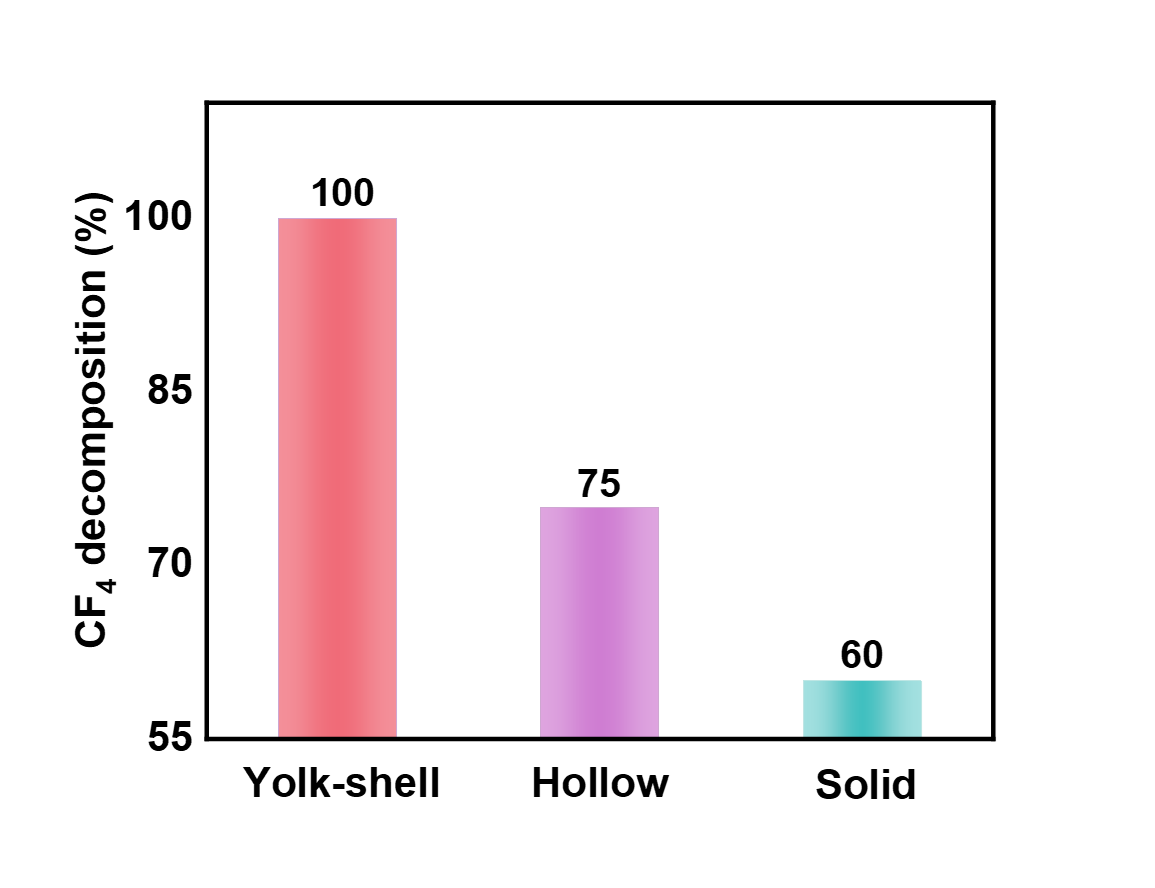


**Figure S14.** CF_4_ decomposition of yolk-shell, hollow and solid Al_2_O_3_ catalysts at 580 ^o^C.


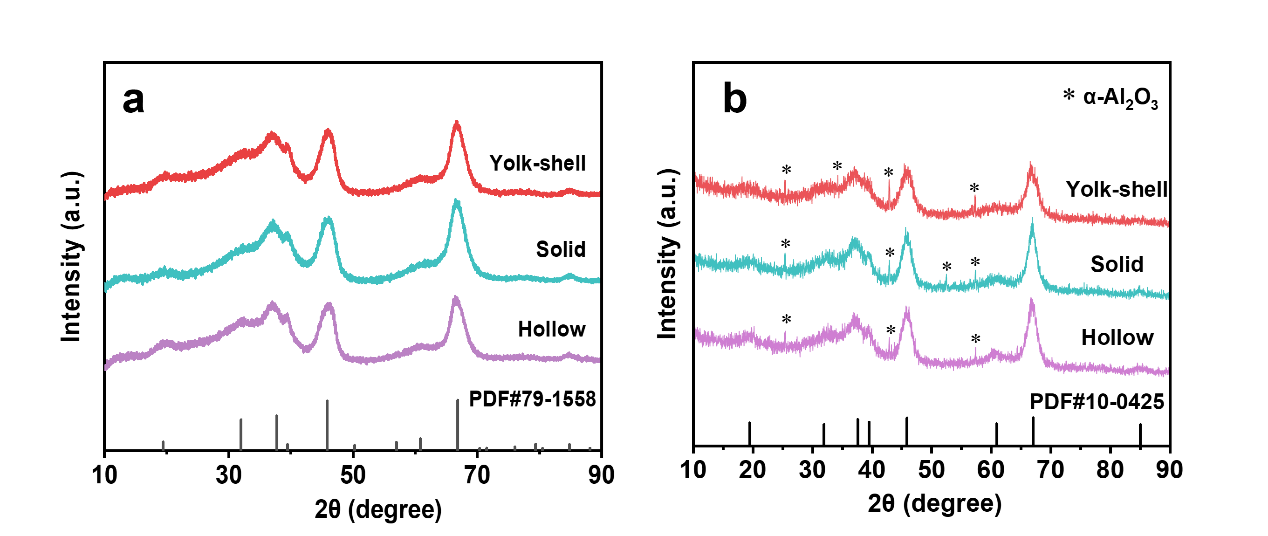


**Figure S15.** XRD patterns of the yolk-shell, hollow, solid Al_2_O_3_ catalysts. (a) before use; (b) after use.


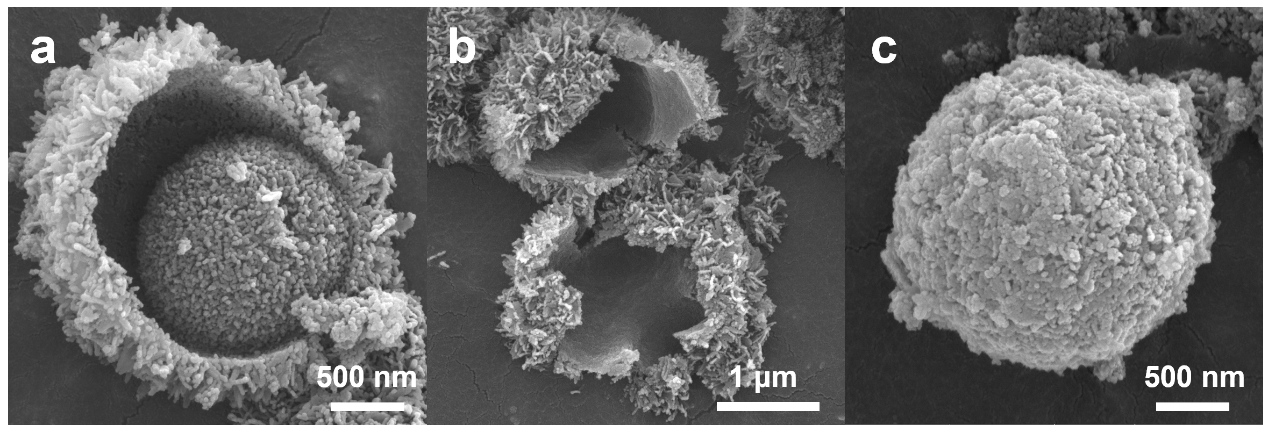


**Figure S16.** SEM images of the three catalysts after the reaction. (a) yolk-shell; (b) hollow;(c) solid Al_2_O_3_ catalysts.

Table S1. BET results of yolk-shell, hollow and solid Al_2_O_3_ catalysts.

| **Samples** | **SA (m^2^ g^-1^)** | **PV (cm^3^ g^-1^)** | **APR (nm)** |
| --- | --- | --- | --- |
| Yolk-shell Al_2_O_3_ | 305.3 | 1.5 | 19.7 |
| Hollow Al_2_O_3_ | 331.4 | 1.2 | 13.9 |
| Solid Al_2_O_3_ | 283.6 | 1.3 | 18.6 |

Table S2. Turnover frequency of the CF_4_ decomposition at 500 °C.

| **Sample** | **surface Al_Ⅲ_ *^a^* (μmol)** | **CF_4_ decomposition *^b^* (%)** | **TOF *^c^* (10^-3^ s ^-1^)** |
| --- | --- | --- | --- |
| Yolk-shell Al_2_O_3_ | 4.67 | 33.46 | 4.44 |
| Hollow Al_2_O_3_ | 4.71 | 23.07 | 3.03 |
| Solid Al_2_O_3_ | 4.61 | 12.33 | 1.66 |

a Determined by the NH_3_-TPD and Py-IR result.

b CF_4_ decomposition at 500 °C (2.0 g catalyst, 2500 ppm of CF_4_ and 10% of H_2_O balanced with Ar, 33.3 mL min^-1^).

c Turnover frequency of the CF_4_ decomposition at 500 °C.

Calculation equation: TOF=$\frac{\text{[CF}_{\text{4}}\text{ flow rate (}\text{μmol}\text{ }\text{s}^{\text{-1}}\text{)]×}\text{[}\text{CF}_{\text{4}}\text{ decomposition]}}{\text{surface }\text{Al}_{\text{III}}}$

**Table S3.** Comprehensive comparison of activity parameters for CF_4_ catalytic hydrolysis based on reported results.

| **Catalysts** | **Reaction temperatures (℃)** | | **Decomposition (%)** | **Lifetime**  **(h)** | **Ref.** | |  |
| --- | --- | --- | --- | --- | --- | --- | --- |
| Yolk-shell Al_2_O_3_ | 580 | | 100 | 150 | This work |  |  |
| S-Al_2_O_3_@ZrO_2_ | 580 | | 100 | 10 | *Proc. Natl. Acad. Sci. U.S.A.* **2023**, *120*, e2312480120 |  |  |
| S-ZrO_2_ | 650 | | 100 | — | *Environ. Sci. Nano*, **2024**, *11*, 881 |  |  |
| Hf–Al_2_O_3_ | | 650 | 100 | 17 | *Environ. Sci. Nano*, **2023**, *10*, 3307 |  |  |
| γ-Al_2_O_3_ | | 650 | 100 | 2 | *Environ. Sci. Nano* **2022**, *9*, 954 |  |  |
| Zr/γ-Al_2_O_3_ | | 650 | 85 | 60 | *Catalysts* **2022**, *12*, 313 |  |  |
| CaO/zeolite | | 650 | 90 | 5 | *J. Environ. Chem. Eng.* **2020**, *8*, 103763 | | |
| S/Ce/γ-Al_2_O_3_ | | 650 | 50 | 50 | *J. Mol. Catal. A Chem.* **2013**, *370*, 50 |  |  |
